# The complete chloroplast genome of *Damnacanthus indicus* C.F.Gaertn. (Rubiaceae)

**DOI:** 10.1080/23802359.2021.1903365

**Published:** 2021-03-28

**Authors:** Shu-Mao Lou, Hong Li, Xin-Mei Qin, Zhang-Ping Huang, Shui-Yuan Jiang, Xi-Yang Huang

**Affiliations:** a Key Laboratory of Ecology of Rare and Endangered Species and Environmental Protection, Ministry of Education, Guangxi Normal University, Guilin, China; b Guangxi Key Laboratory of Rare and Endangered Animal Ecology, College of Life Science, Guangxi Normal University, Guilin, China; c Guangxi Key Laboratory of Functional Phytochemicals Research and Utilization, Guangxi Institute of Botany, Guangxi Zhuang Autonomous Region, Chinese Academy of Sciences, Guilin, China

**Keywords:** *Damnacanthus indicus*, chloroplast genome, phylogeny

## Abstract

*Damnacanthus indicus* C.F.Gaertn. is an understorey shrub widely distributed in East Asia. In this study, the complete chloroplast genome of *D. indicus* was assembled and annotated. The chloroplast genome is 153,997 bp in total length, consisting of a large single-copy region (LSC 85,159 bp), a small single-copy region (SSC 17,584 bp) and two inverted repeat regions (25,627 bp for IRA and IRB,respectively). It contains 134 genes, including 89 protein-coding genes, 37 tRNA genes, and 8 rRNA genes. The phylogenetic analysis indicated that *D. indicus* is sister to *Mitchella repens*, suggesting a close relationship of the two genera.

*Damnacanthus indicus*, an evergreen shrub in the forest understorey is widely distributed in East Asia (Chen et al. 2011). Nothing about the whole chloroplast genome of the species and the corresponding genus has been known yet. In the present study, We report the complete chloroplast genome of *D. indicus* to better understand the structure and the phylogenetic relationship of *D. indicus* as well as *Damnacanthus* to the related taxa.

The samples were collected from Lushan, Jiangxi, China (29°32′28″N, 115°54′47″E) and the voucher specimen was deposited at the Herbarium (IBK) of Guangxi Institute of Botany, Guangxi Zhuang Autonomous Region and Chinese Academy of Sciences (specimen code: IBK00430864). The total DNA was extracted from the silica-dried leaves of *D. indicus* using a modified CTAB method (Doyle and Doyle 1987) and sequenced by the Illumina HiSeq 2500 platform. Approximate 20.2 GB high-quality clean reads were obtained after filtering. The complete chloroplast genome was assembled using GetOrganelle (Jin et al. 2020) and annotated using PGA (Qu et al. 2019) and the online tool GeSeq (Tillich et al. 2017) with reference to the chloroplast genome of *Morinda officinalis* (NC028009) and *Mitchella repens* (KY378710). And the sequence was submitted to the GenBank (accession number: MW548283).

The complete chloroplast genome of *D. indicus* is 153,997 bp in length, consisting of a large single-copy region (LSC 85,159 bp), a small single-copy region (SSC 17,584 bp), and two inverted repeat regions (IRA and IRB 25,627 bp). The complete chloroplast genome contains 134 genes, including 89 protein-coding genes, 37 transfer RNA (tRNA) genes, and 8 ribosomal RNA (rRNA) genes. Among them, 21 genes contain one intron, while 2 genes have two introns. The GC content of the chloroplast genome is 37.8%.

To investigate the phylogenetic position of *D. indicus*, maximum likelihood (ML) phylogenetic tree was constructed using RAxML (Stamatakis 2014) at the CIPRES website (Miller et al. 2010) based on 15 chloroplast genomes of Rubiaceae (Figure 1). The GTRGAMMA substitution model was selected and 1000 bootstrap pseudoreplications were conducted with all other parameters kept as default. It showed that *Damnacanthus* is sister to *Mitchella* with 100% bootstrap support.

**Figure 1.**
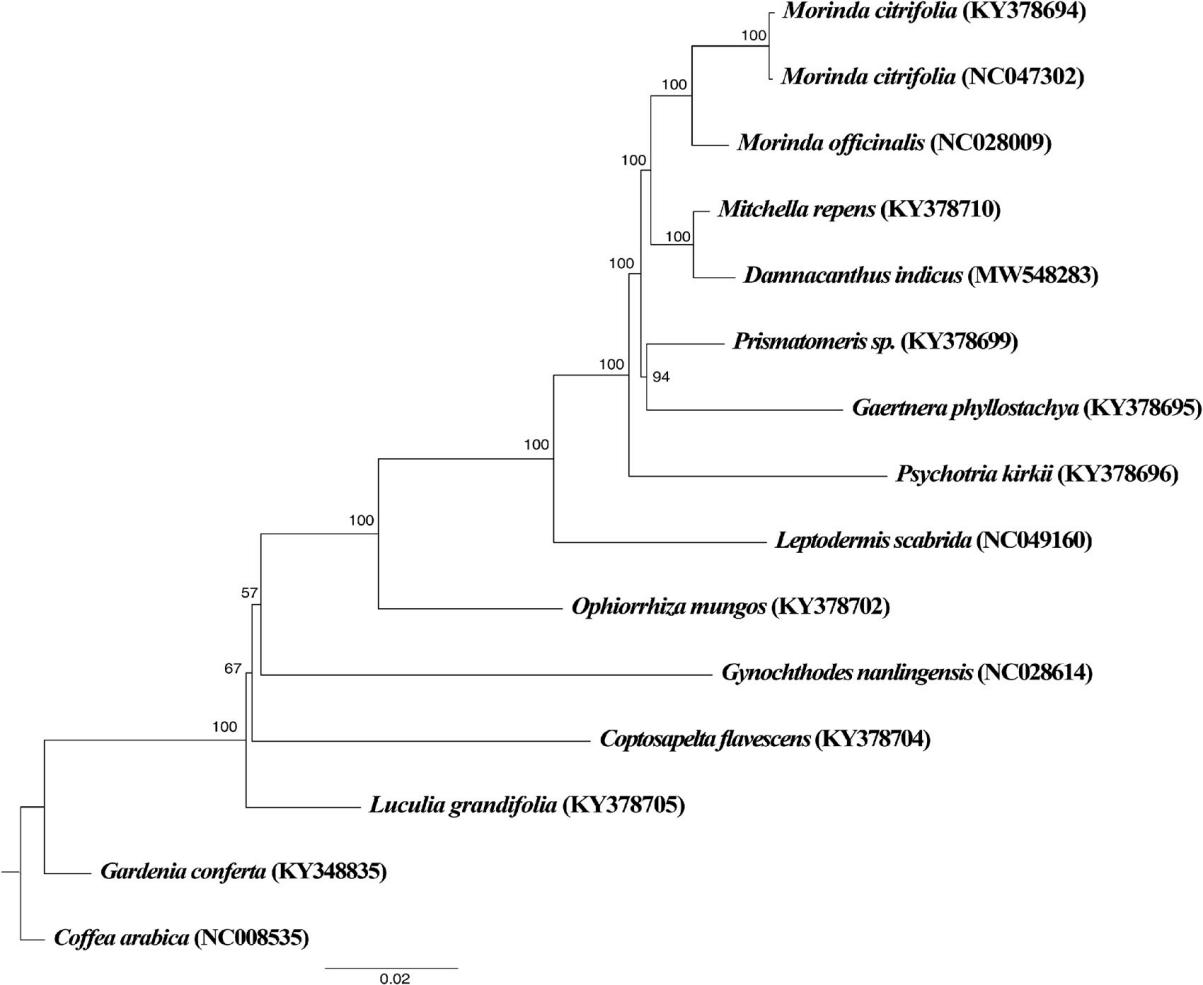
The ML tree showing the phylogenetic placement of the genus *Damnacanthus* represented by *D. indicus*. Bootstrap support is given for each of the branches.

## Data Availability

The genome sequence data that support the findings of this study are openly available in GenBank of NCBI at (https://www.ncbi.nlm.nih.gov/) under the accession no. MW548283. The associated BioProject, SRA, and Bio-Sample numbers are PRJNA706503, SRR13853872, and SAMN18133720, respectively.
